# Modelling the Impact of Antiretroviral Use in Resource-Poor Settings

**DOI:** 10.1371/journal.pmed.0030124

**Published:** 2006-03-14

**Authors:** Rebecca F Baggaley, Geoff P Garnett, Neil M Ferguson

**Affiliations:** **1**Department of Infectious Disease Epidemiology, Imperial College London, United Kingdom; Harvard School of Public HealthUnited States of America

## Abstract

**Background:**

The anticipated scale-up of antiretroviral therapy (ART) in high-prevalence, resource-constrained settings requires operational research to guide policy on the design of treatment programmes. Mathematical models can explore the potential impacts of various treatment strategies, including timing of treatment initiation and provision of laboratory monitoring facilities, to complement evidence from pilot programmes.

**Methods and Findings:**

A deterministic model of HIV transmission incorporating ART and stratifying infection progression into stages was constructed. The impact of ART was evaluated for various scenarios and treatment strategies, with different levels of coverage, patient eligibility, and other parameter values. These strategies included the provision of laboratory facilities that perform CD4 counts and viral load testing, and the timing of the stage of infection at which treatment is initiated. In our analysis, unlimited ART provision initiated at late-stage infection (AIDS) increased prevalence of HIV infection. The effect of additionally treating pre-AIDS patients depended on the behaviour change of treated patients. Different coverage levels for ART do not affect benefits such as life-years gained per person-year of treatment and have minimal effect on infections averted when treating AIDS patients only. Scaling up treatment of pre-AIDS patients resulted in more infections being averted per person-year of treatment, but the absolute number of infections averted remained small. As coverage increased in the models, the emergence and risk of spread of drug resistance increased. Withdrawal of failing treatment (clinical resurgence of symptoms), immunologic (CD4 count decline), or virologic failure (viral rebound) increased the number of infected individuals who could benefit from ART, but effectiveness per person is compromised. Only withdrawal at a very early stage of treatment failure, soon after viral rebound, would have a substantial impact on emergence of drug resistance.

**Conclusions:**

Our analysis found that ART cannot be seen as a direct transmission prevention measure, regardless of the degree of coverage. Counselling of patients to promote safe sexual practices is essential and must aim to effect long-term change. The chief aims of an ART programme, such as maximised number of patients treated or optimised treatment per patient, will determine which treatment strategy is most effective.

## Abstract 2

Modelling results suggest that antiretrovirals will not work as direct prevention tools, and that concurrent promotion of safe sex practices is essential to prevent increased transmission.

## Introduction

Antiretroviral therapy (ART) has greatly reduced HIV/AIDS-related mortality and morbidity in industrialised countries [ 1]. Because it reduces a patient's viral load, it is also believed to reduce infectiousness [ 2], and so has been suggested as a prevention tool in its own right, as well as a treatment [ 3]. As prevention efforts in resource-poor settings have not always met with success, especially in high-prevalence areas of Africa [ 4, 5], this could be an important additional goal of the accelerated roll-out of ART across the continent. In order to maximise the benefit of ART to patients and their communities, its impact on HIV epidemics should be evaluated, and different approaches to ART delivery should be investigated.

The effect that ART will have on transmission will rely not only on its effect on life expectancy, infectiousness, and treatment failure rates, but also on the stage of infection at which treatment is initiated, levels of coverage (which rely on the availability of resources and identification of patients qualifying for ART), and the scale and stage of HIV epidemic that the community is experiencing.

Previous mathematical models have predicted that ART will have a striking effect on transmission when coverage is high. Models based on the HIV epidemic among men who have sex with men in San Francisco [ 6] and Australia [ 7] have produced optimistic outcomes, provided that risk-taking behaviour (e.g., sexual partner change rate) does not increase substantially. However, it is unlikely that treatment of patients in resource-poor settings will be at an early stage of infection (i.e., before CD4 cell counts reach 200 cells/ml or less); many are diagnosed only when their CD4 counts have reached less than 50 cells/ml [ 8, 9]. Models need to explore more realistic patterns of ART use in such settings, incorporating the problems of late presentation of HIV infection, rationing of finite resources, limited health care facilities, and inadequate nutrition.

Among the issues that need addressing are inclusion and exclusion criteria for ART programmes, which may involve tuberculosis status (and with that perhaps a proven record of good adherence from a Directly Observed Therapy Short-Course, or DOTS, programme) and infection stage. ART may be targeted at patients with AIDS, those most in need and closest to death, or it may also be used for those with “pre-AIDS” (defined as the disease state at which the virus escapes immune system control, and CD4 counts decline, but no AIDS-defining conditions [ADCs] appear). Patients treated at the pre-AIDS stage are likely to have a better prognosis than those treated after they have developed full AIDS, because of preserved immune function, and they are likely to avoid the complications associated with immune reconstitution syndrome [ 10]. Given that data are limited on effectiveness of ART programmes in resource-poor settings, further investigation of possible optimal approaches—to include cost-effectiveness analyses—would be valuable before programme roll-out [ 11].

Mukherjee et al. [ 12] believe that the implementation of ART cannot wait until sufficient infrastructure is in place, and they discuss the problem of limited laboratory infrastructure, suggesting that a stepwise progression as more resources become available would be appropriate. They recommend initially implementing an ART programme based on clinical management (or at most total lymphocyte count), with sequential addition of CD4 count testing, viral load testing, and resistance testing. Our analysis investigated the effect of sequential adoption of these laboratory facilities on key outcome measures. The adoption of CD4 count testing allows for the identification of infected individuals who present without an ADC, but are still at a relatively late stage of infection, with a CD4 count in decline (pre-AIDS patients). The adoption of viral load testing can assist with the identification of such individuals, but additionally can identify treatment failure with viral rebound, which can precede by a substantial period a further decline in CD4 count or a reappearance of opportunistic infections [ 13– 15]. However, where funds for HIV/AIDS diagnosis and treatment are limited, the expense of such facilities restricts the number of patients being treated. Furthermore, these facilities allow the identification of individuals who are more likely (pre-AIDS patients) and less likely (those failing therapy) to benefit from ART, leading to difficult ethical considerations concerning prioritisation.

In the current study, we explore many of these issues by predicting and comparing through modelling the epidemiological impacts of alternative strategies, and in the process provide an illustration of the trade-offs involved.

## Methods

### Model and Assumptions

**Figure 1:**
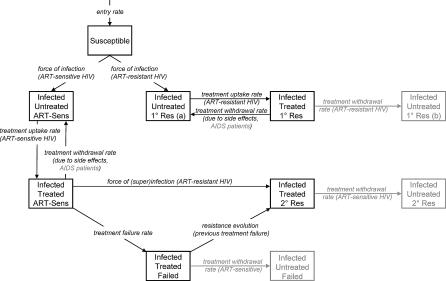
Schematic Illustration of the Structure of the HIV Transmission Model For clarity, stages of infection (primary infection, incubation, pre-AIDS, and AIDS) and death rates are not shown. “1° Res” denotes those with primary (transmitted) resistance, while “2° Res” denotes those with secondary (acquired) resistance. “ART-Sens” denotes people infected with ART-sensitive virus. Treatment withdrawal, used in some scenarios, is shown in grey.

A compartmental deterministic mathematical model describing HIV transmission and ART use was constructed. The basic structure of the model is illustrated in Figure 1 and described in more detail in Protocol S1. The model tracks two HIV strains, one resistant to ART and one sensitive. Each compartment of infected individuals has a defined infectiousness determined by treatment status and viral resistance type. Superinfection is incorporated only for individuals undergoing successful treatment for an ART-sensitive strain, where superinfection with an ART-resistant strain can occur. It is assumed that treatment failure (defined as viral rebound or failure to achieve viral suppression) precedes resistance evolution.

#### ART

We assumed a single, standard triple-combination therapy regimen, with no second-line or salvage therapy available for those who experience treatment failure. The World Health Organisation guidelines for ART use in resource-poor settings recommend at least one combination salvage therapy option [ 8], which may be feasible and could be investigated by extending this model. However, as this may not be realistic in all regions, especially where access to first-line therapy must be rationed, we believe that the assumption of one ART regimen only is an appropriate starting point.

#### HIV and AIDS

In the model, an ART-resistant strain is resistant to the triple regimen rather than to individual drugs within that regimen. It is assumed that there is no pre-existing background level of resistance in the population generated by ART drug use outside the modelled ART programme.

The model was solved numerically using the Runge-Kutte 4 algorithm in Berkeley Madonna version 8.0.1, but programmed concurrently using C for validation.

The model was stratified into four distinct HIV infection stages with different associated infectiousness: primary infection, incubation, pre-AIDS, and AIDS. The incubation period is further divided into eight compartments, with an average duration in each of one year, in order to approximate the average time spent in this stage more accurately than by using an exponential distribution.

#### Sexual behaviour and treatment outcomes

Sexual partnerships in this type of model have no duration—they form (and implicitly end) instantaneously, accompanied by infection transmission, if this has been determined to occur. The model assumes that all partnerships are heterosexual. It does not distinguish between the sexes, but includes four sexual activity classes. A small proportion of the population (0.1%) is in the highest activity class; 26%, 59%, and 15% make up the respective lower-activity classes, with partner change rates of 153, 13.6, 0.5 and 0.2 partners per year, respectively. These form sexual activity groups 1 to 4, respectively. All individuals progressing to AIDS move to the least-active group because of their illness.

Patients starting ART spend an average of six months in an “initial” compartment. For AIDS patients in this “initial” period, the assumed effects of ART on sexual activity are not yet imposed—they have not yet sufficiently recovered to increase any risk-taking behaviour. For pre-AIDS patients, with milder disease, an immediate decrease in sexual activity (dropping to the next lowest sexual activity class) is assumed to follow diagnosis and effective counselling. The incorporation of these compartments allows for different treatment outcomes for AIDS and pre-AIDS patients initiating ART.

On leaving this “initial” phase, a fraction of patients are assumed to drop out (due to tolerability problems) and return to the “untreated” compartment at the same infection stage. The fraction that drops out is higher for AIDS patients than for pre-AIDS patients, because of immune reconstitution syndrome. A substantial proportion of treated AIDS patients die after leaving this stage, an assumption that reflects the high mortality observed in the first few months of treatment for those at late-stage infection [ 16]. All other patients move to “successfully treated” or “unsuccessfully treated” compartments, from which point patients initiating ART at AIDS and pre-AIDS have the same progression rates. A fraction (85%) of AIDS patients increase sexual activity after initiation of ART and move to the next higher sexual activity group. For simplicity, this effect does not wane with time. These are the baseline assumptions regarding changes in the risk-taking behaviour of HIV-infected patients upon initiating ART.

Resurgence of clinical symptoms was modelled as progression through eight treatment stages without symptoms before development of a new ADC upon entering the ninth compartment, when all patients revert back to the lowest sexual activity group. “Successful” treatment is defined as long-term viral suppression, while “unsuccessful” treatment is viral outgrowth (treatment failure). A fraction of patients fail treatment each year, from which point their progression through the stages to AIDS is faster. Patients may also develop resistance as a result of treatment failure or become superinfected with ART-resistant virus. In the case of primary resistance, there is also staged progression back to AIDS upon treatment initiation, but for simplicity, there is no “initial” period.

#### Parameter values

**Figure 2:**
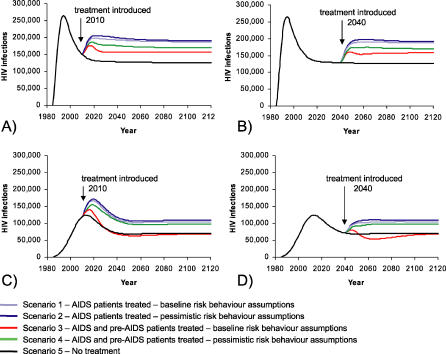
Total Number of HIV Infections through Time for Various HIV Epidemics and under Different Assumptions regarding Behaviour Change of Treated Patients (A) and (B) illustrate a high prevalence, mature epidemic, similar to that of Malawi; (C) and (D) a smaller-scale epidemic (partner change rates one-third of those for the Malawi simulation). (A) and (C) introduce ART in 2010, before equilibrium for (A) and early in the epidemic for (C); (B) and (D) introduce ART in 2040, once equilibrium is reached for (B) and after the peak of the epidemic for (D). For each HIV epidemic scenario graph, two treatment options and two behaviour change scenarios are illustrated: (1) treatment of AIDS patients only, baseline behaviour change assumptions ( Table 3); (2) treatment of AIDS patients only, pessimistic behaviour change assumptions ( Table 3); (3) treatment of AIDS and pre-AIDS patients, baseline behaviour change assumptions; (4) treatment of AIDS and pre-AIDS patients, pessimistic behaviour change assumptions.

**Figure 3:**
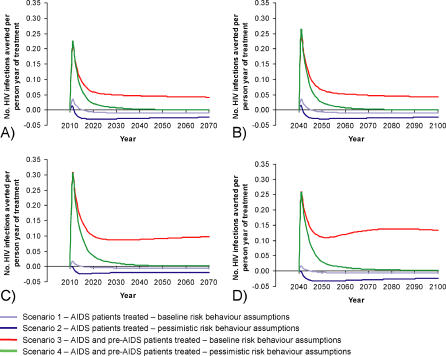
Total Number of HIV Infections Averted per Person-Year of Treatment for Various HIV Epidemics and under Different Assumptions regarding Behaviour Change of Treated Patients Scenarios as for Figure 2.

**Figure 4:**
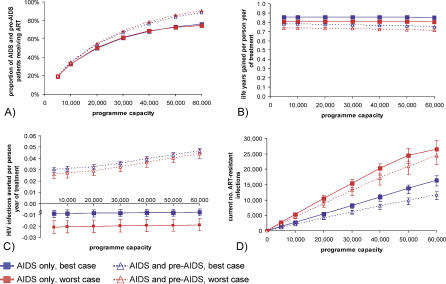
Effect of Programme Capacity Outcome measures ten years after introduction of ART compared with no treatment, for various levels of coverage (ART programme capacities of 5,000 to 60,000) and for treating AIDS patients only or AIDS and pre-AIDS patients. Results are for the epidemic calibrated to Malawi prevalence data. Best- and worst-case scenarios refer to optimistic or pessimistic outcomes of ART use, respectively (see Table 1). Shown are coverage level, defined as proportion of individuals in need (AIDS and pre-AIDS patients) receiving ART (A); cumulative number of life-years gained over ten years, per person-year of treatment (B); HIV infections averted per person year of treatment over ten years (C); and current number of ART-resistant infections ten years after ART introduction (D). Results are the median values of the LHS sensitivity analysis; error bars represent the interquartile range.

**Figure 5:**
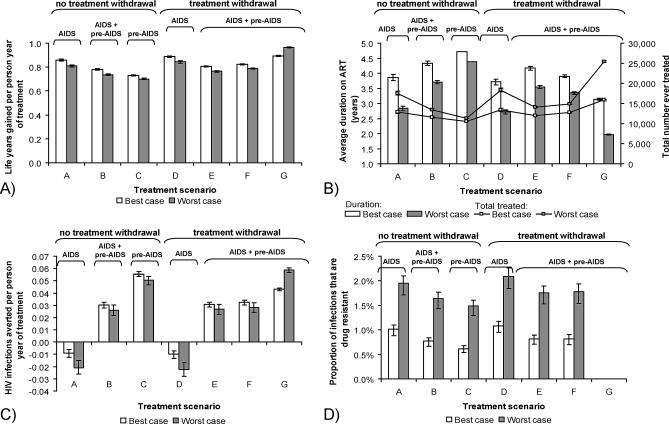
Description of Potential Treatment Strategies for ART Programmes in Resource-Limited Settings Types of ART programme may vary by availability of laboratory tests (CD4 count and viral load testing) and approach to rationing (preferentially treating those at different stages of infection and possibly withdrawing failing treatment). Different strategies are simulated by varying which populations qualify for treatment, and withdrawal of therapy after viral rebound, immunologic, or clinical treatment failure. Outcome measures are ten years after introduction of ART compared with no treatment. The seven scenarios, labelled A–G on the *x*-axes of the bar graphs, refer to different levels of use of laboratory testing and prioritisation of different groups of patients (see Table 4). Best and worst cases refer to optimistic and pessimistic assumptions regarding ART (see Table 1). Shown are cumulative life-years gained per person-year of treatment compared to no treatment (A); average duration on ART per patient and cumulative number ever treated (B); HIV infections and deaths (all causes) averted per person-year of treatment compared to no treatment (C); and proportion of all infections in the population that are ART-resistant at ten years after ART introduction (D). Results are the median values of the LHS sensitivity analysis; error bars represent the interquartile range.

The majority of parameter values were selected after a review of literature and are presented in Table 1 and Table S1. The baseline values were used for model runs in Figures 2 and 3, and the ranges were used for the uncertainty analyses employed in Figures 4 and 5. Table 1 illustrates the range of parameter values used for Latin hypercube sampling (LHS) [ 17] and for the best- and worst-case scenarios for ART, both used in Figures 4 and 5. LHS parameters were varied independently of one another. “Best-” and “worst-case” scenarios in Table 1 refer to the effectiveness of ART provision in terms of mortality and drop-out rates of AIDS patients in the initial treatment period and treatment failure for all ART recipients. This is to reflect the success of the programme in promoting adherence, ensuring adequate drug supplies, managing complications, and providing other medications and nutritional supplements where necessary. A best-case scenario would have low rates of treatment failure, because patient adherence is high and drop-out low. A full listing of all model parameters and additional reference sources is provided in Table S1.

**Table 1:**
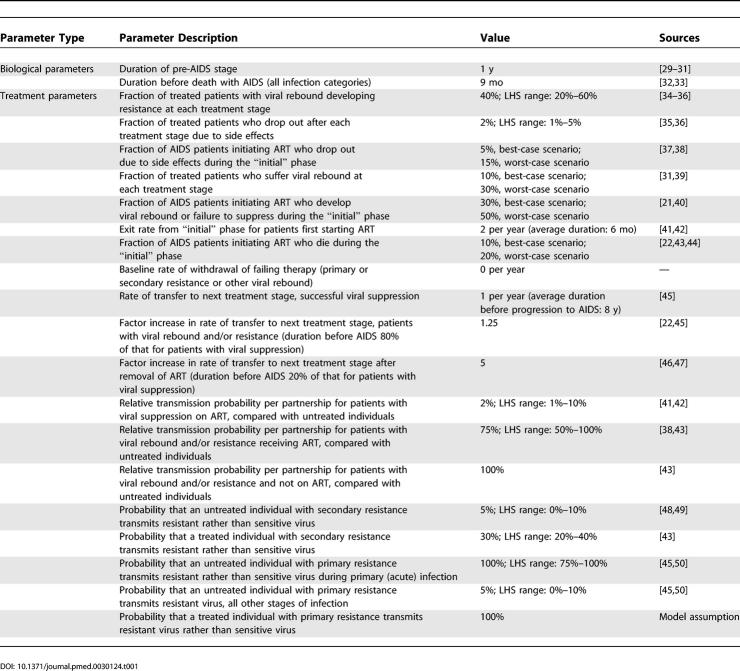
Descriptions and Baseline Values of Some Key Model Parameters, with Sources

Reliable estimates for partner change rates and HIV infectiousness are difficult to obtain [ 18, 19]. Parameter values were chosen to represent the HIV epidemic in Malawi as closely as possible, using data on HIV prevalence from antenatal clinics in major urban areas [ 20]. Malawi was chosen as an example of a country with a mature, high-prevalence HIV epidemic and limited resources with which to provide ART for all those in need. These values also reflect the situation in resource-poor settings more generally. Partner change rates, distributions of individuals between sexual activity groups, and degree of assortative mixing between these groups were parameterised by fitting the model to the prevalence data using the curve fit function in Berkeley Madonna, having seeded the population with five infected individuals with primary infection in the highest sexual activity class (we consider a hypothetical ART programme in a population of one million). The partner change rate for individuals in the highest-risk group is set very high, at 153 partners per year, while that of the lowest-risk group is just 0.23 partners per year, in order to mimic the timing and scale of the Malawi epidemic as closely as possible, with a peak prevalence of 27%. There are large differences in HIV prevalence between major urban and non-urban areas in Malawi [ 20]. We calibrated the model to urban prevalence, as it is likely that ART will be introduced in urban areas before gradual roll-out to other regions. A graph comparing the model simulation run using the parameters in Table 1 with the antenatal clinic prevalence data from Malawi is shown in Figure S1.

Life-years gained for each scenario were calculated by integrating the total number alive and comparing with the no-treatment scenario. Person-years of treatment were calculated by integrating the total number on treatment.

### Analyses of ART Effect

#### Effect of ART on different HIV epidemics

In order to model different types of HIV epidemics, with different basic reproductive numbers and thus scales, the partner change rates across all activity groups were cut to one-third of their original value, generating an epidemic with a peak prevalence of 14.4%. ART was introduced in 2010 and 2040 to investigate the impact by maturity of each epidemic, with unlimited treatment of eligible individuals (details are provided in Table 2). HIV patients are recruited into the ART programme very quickly (an average of one month after qualifying for treatment due to reaching the appropriate HIV infection stage). This represents the most optimistic scenario, in which all individuals qualifying for ART are screened, identified, and treated virtually immediately, in order to investigate the maximum impact of ART. Results are investigated with baseline assumptions regarding behaviour change upon initiating ART, as described above, compared with more pessimistic assumptions, as described in Table 3.

**Table 2:**
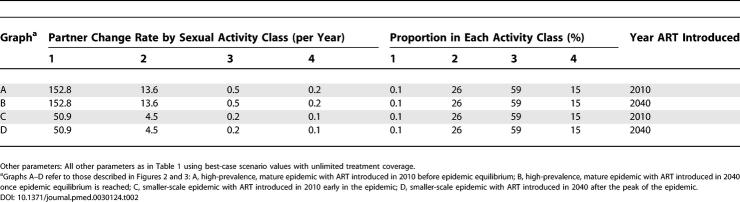
Summary of Treatment Scenarios—Effect of ART on Various Epidemics: Changes in Size and Maturity of HIV Epidemic

**Table 3:**
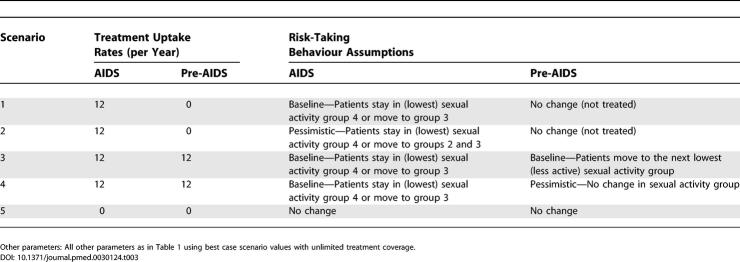
Summary of Treatment Scenarios—Effect of ART on Various Epidemics: Changes in ART Patients' Risk-Taking Behaviours

**Table 4:**
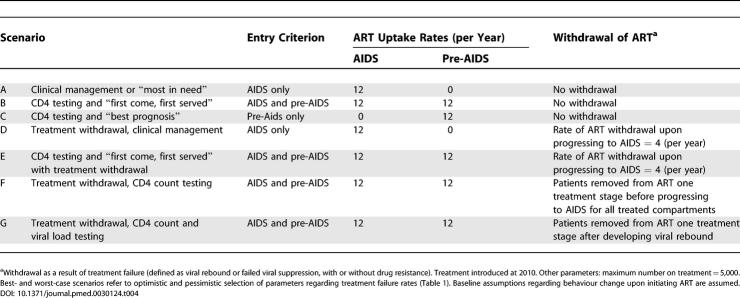
Summary of Treatment Scenarios—Availability of Laboratory Facilities

#### Impact of ART—Levels of coverage

Using baseline values for all parameters ( Table 1) and for baseline changes in risk-taking behaviour upon initiating ART, key outcome measures were recorded for ART programme capacities ranging from 5,000 to 60,000 patients. Best- and worst-case scenarios as well as scenarios in which AIDS patients only versus AIDS and pre-AIDS patients qualified for treatment were calculated. ART was introduced in 2010 with an uptake rate of 12, which equates to uptake of ART an average of one month after progressing to the eligible treatment stage, either AIDS or pre-AIDS.

#### Impact of ART—Treatment strategies

To represent the availability or absence of CD4 count testing and viral load monitoring, timing of treatment initiation, and withdrawal of failing treatment, parameters were varied as described in Table 4. The model assumes that without CD4 count or viral load testing, only AIDS patients (defined as those showing ADCs) can be identified and treated (scenarios A and D; Table 4). A programme that offers CD4 count testing can treat individuals at an earlier stage of immunologic decline, at the pre-AIDS stage (scenarios B and C, E–G; Table 4). Where drugs are rationed, we explored two possible approaches: treatment in the order in which patients present to the clinic (a “first come, first served” approach, scenario B; Table 4), or preferential treatment of patients most likely to benefit from ART and least likely to suffer complications such as immune reconstitution syndrome and treatment failure (a “best-prognosis” approach, scenario C; Table 4).

Treatment failure in this case was defined as viral rebound, which can often precede immunologic treatment failure (decline in CD4 count) and clinical progression by a considerable period [ 15], with patients still able to respond well to ART [ 21, 22]. Scenario D investigates clinical management, where patients having failed ART are removed from therapy an average of three months after developing AIDS. In scenario E, although CD4 testing would allow earlier detection of immunologic treatment failure, it is still assumed that ART is withdrawn only from those with AIDS, whereas the earlier withdrawal due to the detection of failure is used in scenario F. Scenario F involves withdrawal of ART at this detection stage, when immunologic decline is identified. ART is withdrawn from patients one treatment stage before progressing to AIDS, i.e., within a year of developing a new ADC.

Scenario G incorporates viral load testing and assumes the extreme measure of withdrawing all ART at the earliest definition of treatment failure, viral rebound, when patients are still benefiting from treatment. Viral load testing is assumed to identify treatment failure and facilitate withdrawal of individuals from therapy less than one year after treatment failure.

## Results

### Effect of ART on Various HIV Epidemics

The scenarios in Figures 2 and 3 illustrate the various long-term impacts that ART may have on HIV prevalence when its availability is not limited. The impact of widespread ART on the HIV epidemic ( Figure 2) and cumulative HIV infections averted per person-year of treatment ( Figure 3) is illustrated. All scenarios have high treatment uptake rates, requiring a rigorous screening programme in order to identify all patients eligible for ART, and the best-case scenarios are used. Therefore this result represents the best possible outcome of ART introduction.

The impacts of treating AIDS patients and pre-AIDS patients differed, partly because, in these unlimited-treatment scenarios, the numbers receiving ART differed substantially. Treating solely AIDS patients increased prevalence for all modelled epidemics by increasing life expectancy, while additionally treating pre-AIDS patients initially counteracted this increase to some extent by averting more infections ( Figure 3). Changing the assumptions regarding behaviour change upon initiation of ART (see Table 3 for details) had little effect when ART eligibility was limited to AIDS patients. In contrast, the “pessimistic” assumption of an absence of behaviour change for treated pre-AIDS patients (scenario 4; Table 3) substantially increased prevalence for all scenarios, especially for the epidemic with the lower partner change rates and *R _0_* ( Figure 2). The model suggests that the most “optimistic” impact that ART may have on an HIV epidemic with no limitation on numbers treated is no change in number of infections; this scenario would require treatment of all AIDS and pre-AIDS patients, accompanied by significant change in risk-taking behaviour, and an epidemic with an intrinsically low *R _0_* ( Figure 2C and 2D). Figure 3 suggests that, although treating pre-AIDS patients may prevent infections in the short term, over the long term the net gain is considerably reduced. This is not due to outgrowth of ART-resistant viruses; the constraint that wild-type is more transmissible than the resistant phenotype prevents substantial primary resistance under this choice of parameters. Rather, the immediate effect of decreased risk-taking behaviour among treated patients was negated by their increased life expectancy.

### Impact of ART—Levels of Coverage

The effect when there are no constraints on the availability of ART drugs was compared with a limited number being treated at any one time, to reflect resource constraints ( Figure 4). The implication for treatment coverage, in terms of proportion of individuals in need (AIDS and pre-AIDS patients) receiving ART for each programme capacity, is shown in Figure 4A.

Because of the limited effect of treatment on transmission, the benefits of ART to the community per person-year of treatment did not vary substantially with the capacity of a programme, as measured by life-years gained ( Figure 4B). Each additional person treated produced the same benefit in terms of life-years gained and transmission and morbidity reduced—for a mature epidemic, there would be no herd effect producing a non-linear pattern of benefit of increasing access to ART in the short term. Paradoxically, treating both AIDS and pre-AIDS patients saved fewer life-years per person year of treatment than an AIDS-only approach, despite the fact that treating pre-AIDS averted more infections over this period ( Figures 3A and 4C). The benefits of delaying imminent AIDS deaths were demonstrated immediately, while the prevention of future deaths by preventing infection was revealed more slowly. The number of life-years saved per person-year of treatment for pre-AIDS and AIDS treatment overtook that for AIDS-only treatment by 2029 when the model was run with a capacity of 30,000 ART patients and best-case scenario parameters ( Figure S2).

A greater degree of coverage led to a greater degree of viral resistance evolution and potential spread ( Figure 4D). Furthermore, the greater the coverage of a poorly run programme, where resistance evolution is frequent, the greater the opportunity for relatively fit ART-resistant mutants to evolve, which may compromise the entire programme in that area and possibly others.

### Impact of ART—Treatment Strategies

Scenarios that may potentially be adopted by ART programmes in resource-constrained settings are described in Table 4 and Methods (section titled “Impact of ART—Treatment Strategies”), and relate not only to the availability of laboratory facilities but also to the priorities for treatment assigned by public health officials. We assumed that initiating treatment before extensive destruction of the cellular immune system, at pre-AIDS rather than AIDS, leads to less risk of mortality, treatment failure, and dropout due to drug intolerance. We also assumed that treatment failure leads to a decrease in treatment effectiveness, but that ART still has a substantial beneficial effect in the “failed patient” (see Table 1).

Figure 5A again shows that ten years after treatment introduction, the benefits of averting new infections by treating pre-AIDS patients are not evident, so treating only AIDS patients appears to confer more benefit in terms of life-years gained (scenario A versus scenario C). Figure 5B shows that without treatment withdrawal, pre-AIDS patients remained on ART substantially longer than did AIDS patients, because they have a better prognosis (lower mortality and likelihood of treatment failure). The influence of rates of treatment failure on duration of ART is shown by the large differences between best- and worst-case scenarios. Treatment withdrawal significantly decreased the average duration on ART, by over a year in some scenarios. Figure 5C shows that the earlier that individuals were placed on ART, the more infections were averted under these assumptions regarding behaviour change. Figure 5D shows population-level resistance prevalence, which appears relatively low, but was the result of relatively small coverage of the population (approximately 20% of all those in need at 2020).

For treatment withdrawal based on clinical management, there was little impact in terms of infections averted and resistance emergence (scenarios A versus D, and B versus E; Figure 5C and 5D). ART withdrawal was just a few months before death from AIDS, but in the context of rationing, this would free up treatment for others. Therefore, both life-years gained per person-year of treatment and the number of individuals that can be reached with ART increased slightly, because resources would be directed in a more “efficient” way ( Figure 5). However, the difference in life-years gained for all scenarios was small; all patients must receive ART continuously to benefit, so this is not surprising. Assumptions regarding treatment withdrawal for scenario G ( Figure 5) meant that ART was withdrawn from all patients after treatment failure but before emergence of drug resistance; this is unrealistic, but it illustrates the maximum impact that such a policy could have ( Figure 5D). A substantial effect on preventing resistance is observed only for scenario G, with ART withdrawal after viral rebound. Viral rebound can occur shortly after initiating therapy, and potentially years before immunologic decline, while patients are free from symptoms, so risk-taking behaviour could be relatively high and thus there is considerable opportunity for transmission of resistant strains. If withdrawal is only after CD4 counts begin to drop, the chance of resistance transmission is substantially less. The impact on HIV transmission and life-years gained remained minimal ( Figure 5C), and patients' duration on ART (a measure of life expectancy) was substantially reduced, although total numbers benefiting from therapy increased ( Figure 5B).

## Discussion

We have modelled the impact of ART on various types of HIV epidemics in resource-poor settings more realistically than hitherto, to investigate how the specific details of ART programme implementation might affect epidemiological and clinical impact. According to our modelling results, HIV epidemics in sub-Saharan Africa are not amenable to control through treatment, regardless of the extent of ART roll-out, and must be integrated with prevention methods. In the absence of substantial behaviour change of treated patients through effective counselling, prevalence is likely to increase. Different coverage levels for ART would not affect benefits such as life-years gained per person-year of treatment because of the limited effect of treatment on transmission, but increasing coverage does increase the emergence and spread of drug resistance. Scenarios investigating withdrawal of failing treatment increased the numbers who could benefit from AIDS, but effectiveness per person was compromised. Only withdrawal at a very early stage of treatment failure, soon after viral rebound, would have a substantial impact on drug resistance emergence ( Figure 5D), because the majority of transmission of resistant HIV strains would occur before immunologic treatment failure. While our model assumes conservative estimates for the fitness of resistant strains, substantial viral replication under continued treatment pressure could promote generation of even fitter strains, increasing the frequency of resistance transmission events.

While our model incorporated some behaviour change upon initiating ART, it did not consider the complex effects of voluntary counselling and testing on risk-taking behaviour. Both uptake of testing and risk-taking behaviour may vary substantially by scale of coverage and performance of ART programmes, and their impact is likely to have a large effect on prevalence. The model results suggest similar levels of benefit per person-year of treatment, regardless of the scale of ART provision, but in reality, scaling up programmes is likely to compromise quality, meaning higher dropout rates and mortality and treatment failure, negating the beneficial impacts of ART and increasing the rate of drug resistance emergence.

CD4 count testing allows the identification of pre-AIDS patients. Additionally, the implementation of laboratory facilities for viral load monitoring allows the identification of treatment failure earlier, increasing the sustainability of the programme, either by allowing switching to a salvage regimen, or perhaps (in the absence of alternative treatments) by halting therapy and giving the drugs to another patient whose prognosis is better, as is investigated here. The benefits of viral load testing will increase with higher levels of resistance in the population. The model does not incorporate background drug resistance levels due to uses of ART outside the modelled programme, but levels of resistance are likely to be higher in many areas due to prevention of mother-to-child transmission [ 23] and sporadic ART use [ 24], and because some programmes have used only double or single therapy [ 25, 26]. For example, in the UNAIDS/Uganda Ministry of Health Drug Access Initiative pilot project, only 64% of patients were ART drug-naïve before enrolling in the initiative [ 26].

Withdrawal of failing treatment, where this is defined as viral rebound or immunologic failure, when the patient would still benefit from continuing ART, is unlikely to be acceptable to any ethics committee or government. However, the exploration of this strategy through modelling has shown the benefits in terms of increasing access to (albeit less effective) treatment to more individuals, and for withdrawal upon viral rebound, slowing the emergence of drug resistance. While the net health benefits in terms of life-years gained would be similar for each strategy, in a wider context, factors such as delaying orphanhood by even a short time for many patients' children, or by a longer time for fewer, make this comparison difficult to evaluate.

Results from this model suggest that counselling of patients to promote safe sexual practices is essential, and must aim to effect long-term change and prevent behavioural disinhibition (increase in risk-taking behaviour in response to perceptions of safety conferred by the use of ART), not only for ART patients, but for all individuals. Withdrawal of treatment at a potentially acceptable stage, that is, after immunologic deterioration when ART is no longer of benefit, would not have a substantial effect on containing resistance emergence, but would increase the number able to benefit from treatment where its availability is limited. As the pace of ART roll-out across southern Africa quickens, difficult decisions regarding the allocation of finite resources will have to be made. Mathematical models are increasingly being employed to explore optimal strategies for allocation of these funds [ 27, 28]. Analysing the implications of different treatment strategies, using data from early programmes coupled with the types of modelling methods presented here, is needed to give AIDS treatment and prevention programmes the best possible chance of success.

## Supporting Information

Figure S1 Comparison of Modelled Epidemic with Prevalence Data Comparison of the projected epidemic using baseline parameter values, with UNAIDS prevalence estimates from antenatal clinics in Malawi [ 20].
(30 KB PPT).

Figure S2 Comparison of Life-Years Gained per Person-Year of Treatment over Time for AIDS Only and AIDS and Pre-AIDS Treatment Strategies Model run with treatment capacity of 30,000 and best-case scenario parameters.
(39 KB PPT).

Protocol S1 Model Description Detailed description of the model, including transmission equations and a full list of adopted parameter values.
(488 KB DOC).

Table S1 Descriptions and Adopted Values of All Model Parameters (396 KB DOC).

**Patient Summary**

**Background**

Access to antiretroviral therapy (ART) is slowly increasing in resource-poor countries. Strategies for how to achieve the greatest health benefits with what will remain, at least in the near future, limited resources are necessary and being developed. How to distribute a limited amount of life-saving drugs in the face of a devastating pandemic—in other words, how to decide who will not get treatment—is a very difficult ethical question. Those who need to solve it must learn as much as possible from the experiences of others. In addition, theoretical predictions of the outcomes under different scenarios can help. To this end, public health scientists are using mathematical modelling of different scenarios of testing, treatment, and counselling. For the modelling data to be useful, they must be based as much as possible on realistic assumptions.

**Why Was This Study Done?**

Sexual transmission of HIV is more likely if the HIV-positive partner has a higher viral load. Because ART not only slows AIDS progression but also reduces viral load in infected individuals, the drugs therefore not only improve the health and prolong the life of those who take the drugs but also make it less likely that they infect others. As a consequence, ART has been discussed not only as a treatment but also as a prevention tool in its own right. Mathematical modelling supports the notion that ART can reduce transmission rates, as long as the people receiving treatment do not change their sexual behaviour towards riskier sex (unprotected intercourse, more partners, etc.). So far, modelling the prevention effects has been based on experiences in the US and Australia, where most HIV-infected individuals get access to drugs during the early stages of infection, as soon as their CD4 counts drop (this is unlikely to be the case in resource-poor settings where many patients are diagnosed and treated only when they experience AIDS symptoms). These researchers used more relevant parameters to model the effects of ART on improving the health of those treated and on HIV prevalence, i.e., the rate of new infections.

**What Did the Researchers Do and Find?**

They used a model to predict and compare the impacts of alternative strategies. Some of the strategies included the provision of diagnostic laboratories that could routinely measure CD4 counts and viral loads of HIV-infected individuals. Only if this is done could people be treated before they develop overt symptoms. They also took into account different ways that people might change their sexual behaviour if they get treatment (which might make them feel physically better and more likely to be sexually active) and counselling (which will hopefully increase safe sex practices). They found that providing ART to all individuals with AIDS symptoms (i.e., those at the late stages of the disease) was likely to increase the prevalence of HIV infection, as these people live longer and become sexually active again. If ART is also provided to people during the earlier stages of infection, the outcome on HIV prevalence depends on the behaviour of these individuals. If ART was more widely available, the risk of the emergence of drug-resistant strains of the virus increased.

**What Does This Mean?**

These results suggest that provision of ART to symptomatic AIDS patients and/or those at the earlier stages of the disease is not likely to prevent many new infections. It could even increase transmission of the virus as patients live longer and are healthier. Counselling patients and the rest of society to promote safe sex practices must therefore be an essential part of any strategy if it is to contain and reverse the AIDS epidemic. The model presented here can support health policy makers in resource-poor settings in their difficult task of allocating limited amounts of antiretroviral drugs for the greatest benefit of their populations.

**Where Can I Find More Information Online?**

WHO pages on HIV/AIDS:

http://www.who.int/hiv/en

Report from a Consultation on Anti-Retroviral Therapy for HIV Prevention:

http://www.nih.gov/od/oar/public/pubs/art_12_00.htm

Report from a Consultation on Studies of HIV Disease in Developing Countries:

http://www.nih.gov/od/oar/public/pubs/hivdeveloping.htm

Joint United Nations Programme on HIV/AIDS, World Health Organization. AIDS epidemic update 2004:

http://www.unaids.org/wad2004/report.html

Global HIV Prevention Working Group report, HIV Prevention in the Era of Expanded Treatment Access:

http://www.gatesfoundation.org/nr/downloads/globalhealth/aids/PWG2004Report.pdf

## Abbreviations

ADC: AIDS-defining condition
ART: antiretroviral therapy
LHS: Latin hypercube sampling
