# Supplementary material for: Modelling the Impact of Antiretroviral Use in Resource-Poor Settings
Source: PLoS Med. 2006 Mar 14;3(4):e124. doi: 10.1371/journal.pmed.0030124 (PMC1395349; doi:10.1371/journal.pmed.0030124)
Supplement: Supplementary file 1 [file pmed.0030124.sd001.doc]

**Supplementary Material – Model Description**

The state variables and transmission equations for the model are shown below. The sexual activity classes are defined in the model equations below in four groups $i=1..4$, with group 1 having the highest activity and group 4 the lowest. The HIV infection stages are 11 groups $j=1..11$, where $j=1$ is primary infection, $j=10$ is pre-AIDS, $j=11$ is AIDS and the eight groups $j=2...9$, each of mean duration 1 year before progression to the next group, represent the incubation period. The model does not distinguish between genders as this is not required for the research questions being investigated. All individuals progressing to AIDS move to the lowest sexual activity group $i=4$. Treatment of AIDS patients resulting in clinical improvement is denoted by eight treatment stages $j=1...8$, during which a fraction $1-\theta_{4}$ increase their sexual activity by moving to sexual activity group $i=3$. Resurgence of clinical symptoms is modelled as progression back to stage $j=9$, upon which all patients revert back to the lowest sexual activity group. Initiating ART before the AIDS stage results in a decrease in sexual activity; each sexual activity group is moved to the next most active group, in order to reflect counselling and support services provided by the treatment programme.

Superinfection with an ART-resistant strain is possible for treated individuals with viral suppression only, as these are the only individuals without viral outgrowth, and thus will have a pool of target cells rendering them susceptible to re-infection. Rates of treatment failure are determined by parameter $f$. All drug resistance evolves as a consequence of treatment failure, because of viral replication in the presence of continued drug pressure, and is determined by parameter $\kappa$. Table S1 provides a full list of all parameters used in the model with adopted values and sources. Some parameters do not have any sources cited because there is insufficient information available in the literature. Parameter values were chosen to approximate the HIV epidemic in Malawi; a comparison of the model epidemic with actual data is shown in Figure S2.

**State Variables**

For individuals in sexual activity class $i$ and HIV infection stage $j$ (where applicable):

$S_{i}$ = susceptible individuals $\{i=1..4\}$

$I_{Sij}^{U}$= ART-sensitive HIV infected individuals, untreated $\{i=1..4,j=1..11\}$

$I_{Sij}^{T}$= ART-sensitive HIV infected individuals, treated $\{i=1..4,j=1..9\}$

$I_{SPi}^{Temp}$=ART-sensitive HIV infected individuals in the ‘initial’ phase (pre-AIDS patients) $\{i=1..4\}$

$I_{SA}^{Temp}$=ART-sensitive HIV infected individuals in the ‘initial’ phase (AIDS patients)

$I_{Sij}^{TF}$= ART-sensitive HIV infected individuals, treated but viral rebound $\{i=1..4,j=1..9\}$

$I_{Sij}^{UF}$= ART-sensitive HIV infected individuals withdrawn from treatment $\{i=1..4,j=1..9\}$

$I_{R2,ij}^{T}$= ART-resistant HIV infected individuals(secondary resistance), treated $\{i=1..4,j=1..9\}$

$I_{R2,ij}^{U}$= ART-resistant HIV infected individuals (secondary resistance), untreated $\{i=1..4,j=1..9\}$

$I_{R1a,ij}^{U}$= ART-resistant HIV infected individuals (primary resistance), untreated$\{i=1..4,j=1..11\}$

$I_{R1,ij}^{T}$= ART-resistant HIV infected individuals (primary resistance), treated $\{i=1..4,j=1..9\}$

$I_{R1b,ij}^{U}$= ART-resistant HIV infected individuals (primary resistance), withdrawn from treatment

$$\{i=1..4,j=1..9\}$$

**Other values**

$\lambda_{Si}$= force of infection for ART-sensitive virus phenotype

$\lambda_{Ri}$= force of infection for ART-resistant virus phenotype

$N_{i}$= total in sexual activity group $i$

**Transmission equations**

The ordinary differential equations which define the model are shown below.

A description and values for all parameters used in the following equations are given in Table S1.

(1) $S_{i}^{'}=\theta_{i}\chi N_{0}-S_{i}\left(\lambda_{Si}+\lambda_{Ri}+\mu\right)$

(2) $\left\{i=1..4,j=1\right\}$

(3) $\left\{i=1..4,j=2..9\right\}$

(4) $\left\{i=1..4,j=10\right\}$

(5) $\left\{i=1..4,j=11\right\}$

(6) $\left\{i=1..4\right\}$

(7) $\left\{i=4,j=11\right\}$

(8)

$$\left\{i=1..4,j=1\right\}$$

(9) $\left\{i=1..4,j=2..8\right\}$

(10) $\left\{i=1..4,j=9\right\}$

(11) $\left\{i=1..4,j=1\right\}$

(12) $\left\{i=1..4,j=2..8\right\}$

(13)

$$\left\{i=1..4,j=9\right\}$$

(14)

$$\left\{i=1..4,j=1..8\right\}$$

(15) $\left\{i=1..4,j=9\right\}$

(16)

$$\left\{i=1..4,j=1..8\right\}$$

(17) $\left\{i=1..4,j=9\right\}$

(18) $\left\{i=1..4,j=1..8\right\}$

(19) $\left\{i=1..4,j=9\right\}$

(20) $\left\{i=1..4,j=1\right\}$

(21) $\left\{i=1..4,j=2..10\right\}$

(22) $\left\{i=1..4,j=11\right\}$

(23) $\left\{i=1..4,j=1\right\}$

(24) $\left\{i=1..4,j=2..8\right\}$

(25) $\left\{i=1..4,j=9\right\}$

(26) $\left\{i=1..4,j=1..8\right\}$

(27) $\left\{i=1..4,j=9\right\}$

where:

$\varphi_{Sj}^{T}=\varphi_{Sj}^{TF}=\varphi_{Sj}^{U}=\varphi_{Rj}^{T}=\varphi_{Rj}^{U}=0$ $\left\{j=0\right\}$

$\Theta_{i}=0$ $\left\{i=1..3\right\}$ $\Theta_{i}=PZ_{P}I_{SA}^{Temp}$ $\left\{i=4\right\}$ $\left\{j=11\right\}$

$\Lambda_{i}=0$ $\left\{i=1\right\}$ $\Lambda_{i}=\gamma_{Sj}I_{Si-1,j}^{U}$ $\left\{i=2..3\right\}$ $\Lambda_{i}=\gamma_{Sj}\left(I_{Si-1,j}^{U}+I_{Sij}^{U}\right)$ $\left\{i=4\right\}$ $\left\{j=10\right\}$

$K_{i}=0$ $\left\{i=1..2\right\}$ $K_{i}=1-\theta_{4}$ $\left\{i=3\right\}$ $K_{i}=\theta_{4}$ $\left\{i=4\right\}$

$\tau_{i}=0$ $\left\{i=1\right\}$ $\tau_{i}=\gamma_{Rj}I_{R1ai-1j}^{U}$ $\left\{i=2..3\right\}$ $\tau_{i}=\gamma_{Rj}\left(I_{R1ai-1j}^{U}+I_{R1aij}^{U}\right)$ $\left\{i=4\right\}$ $\left\{j=10\right\}$

$H_{xij}^{y}=0$ $\left\{i=1..3\right\}$ $H_{xij}^{y}=\sum_{i=1}^{i=4} I_{xij-1}^{y}$ $\left\{i=4\right\}$ $\left\{j=1..11\right\}$

where:

$$x=S,R1,R1a,R1b,R2$$

$$y=U,T,TF,UF$$

The forces of infection for ART-sensitive ($\lambda_{Si}$) and ART-resistant ($\lambda_{Ri}$) HIV are given below. They are determined by the infectiousness of individuals in each class ($\beta$), the probability that ART-resistant rather than ART-sensitive virus is transmitted in the case of mixed infections ($\omega$), the rate of sexual partner change for individuals in sexual activity group $i$ ($c_{i}$) and the proportion of the total population infected with each strain, contacts with whom are determined by the mixing matrix $\rho_{ik}$. The force of infection is equivalent to the incidence rate per susceptible individual.

(28) $\lambda_{Si}=c_{i}\sum_{k=1}^{k=4} \frac{\rho_{ik}}{N_{k}}\left(\begin{matrix} \sum_{j=1}^{j=11} \left(\beta_{Sj}^{U}I_{Sij}^{U}+\left(\text{1-}\omega_{Uj}^{1}\right)\beta_{Rj}^{U1}I_{R1aij}^{U}\right)+\beta_{S1}^{T}I_{SPi}^{Temp}+\beta_{S9}^{T}I_{SA}^{Temp}+ \\ \sum_{j=1}^{j=9} \left(\begin{matrix} \beta_{Sj}^{UF}I_{Sij}^{UF}\text{+}\beta_{Sj}^{TF}I_{Sij}^{TF}+\beta_{Rj}^{U2}\left[\left(\text{1-}\omega_{U}^{2}\right)I_{R2ij}^{U}+\left(\text{1-}\omega_{Uj}^{1}\right)I_{R1bij}^{U}\right] \\ +\beta_{Rj}^{T}\left[\left(\text{1-}\omega_{T}^{2}\right)I_{R2ij}^{T}+\left(\text{1-}\omega_{T}^{1}\right)I_{R1ij}^{T}\right]+\beta_{Sj}^{T}I_{Sij}^{T} \end{matrix}\right) \end{matrix}\right)$

(29) $\lambda_{Ri}=c_{i}\sum_{k=1}^{k=4} \frac{\rho_{ik}}{N_{k}}\left(\sum_{j=1}^{j=11} \beta_{Rj}^{U1}\omega_{Uj}^{1}I_{R1aij}^{U}+\sum_{j=1}^{j=9} \left(\beta_{Rj}^{U2}\left(\omega_{U}^{2}I_{R2ij}^{U}+\omega_{Uj}^{1}I_{R1bij}^{U}\right)+\beta_{Rj}^{T}\left(\omega_{T}^{2}I_{R2ij}^{T}+\omega_{T}^{1}I_{R1ij}^{T}\right)\right)\right)$

where

(30) $N_{i}=S_{i}+I_{SPi}^{Temp}+B_{i}I_{SA}^{Temp}+\sum_{j=1}^{j=11} I_{Sij}^{U}+I_{Sij}^{T}+I_{Sij}^{TF}+I_{Sij}^{UF}+I_{R2ij}^{T}+I_{R2ij}^{U}+I_{R1aij}^{U}+I_{R1ij}^{T}+I_{R1bij}^{U}$

where $B_{i}=0$ $\{i=1..3\}$ $B_{i}=1$ $\{i=4\}$

**Mixing matrix definition**

$\rho_{ik}$is a mixing matrix, the elements $ik$ of which are the probability that an individual in sexual activity class $i$ will form a partnership with a member of sexual activity class $k$. The value of $\rho_{ik}$ depends on the pattern of mixing which could vary from fully assortative, where partnerships only form within the same activity class, to random, where partnerships form randomly between different activity classes.

(31) $\rho_{ik}=\varepsilon\delta_{lm}+\left(1-\varepsilon\right)c_{k}\frac{N_{k}}{\sum_{i=1}^{i=4} c_{i}N_{i}}$

Where $\delta_{lm}=1$ when $i=j$ and $\delta_{lm}=0$ when $i\neq j$.

Infectiousness of individuals varies with stage of infection. The model assumes that individuals with primary infection and AIDS (stages 1 and 11) have a higher degree of infectiousness than individuals in the incubation period (stages 2 to 9) by factor $a$. For individuals with pre-AIDS (stage 10), it is by factor $a/2$:

(32) $\beta_{xj}^{y}=a\beta_{x}^{y}$ $\{j=1,11\}$

(33) $\beta_{xj}^{y}=\beta_{x}^{y}$ $\{j=2..9\}$

(34) $\beta_{xj}^{y}=\frac{a}{2}\beta_{x}^{y}$ $\{j=10\}$

**Treatment**

ART introduction is delayed until the start time by switching $\gamma_{Sj}$ and $\gamma_{Rj}$ from 0 to their experimental values at time = Start time. For scenarios where a limited number of individuals can receive treatment at one time, the following algorithm was used:

(35) $\gamma_{Sj}=T_{Sj}\left(1-\left(\zeta/\Omega\right)^{5}\right)$

where:

$T_{Sj}$ is the experimental treatment uptake value for those infected with ART-sensitive HIV and at infection stage $j$

$\Omega$ is the maximum number treated

(36) $\zeta=\sum_{j=1}^{j=11} I_{Sj}^{T}+I_{Sj}^{TF}+I_{R2,j}^{T}+I_{R1,j}^{T}$, the total number currently receiving ART.

A sufficiently small step size is required in order to ensure that this algorithm works correctly (0.005).

**Treatment withdrawal**

For scenarios involving withdrawal of ART, the following changes in parameters from baseline values occur:

For withdrawal upon progressing to AIDS (Scenarios D and E):

$\alpha_{Sj}^{T}=\alpha_{Sj}^{TF}=\alpha_{Rj}^{T1}=\alpha_{Rj}^{T2}=4$ year^-1^

For withdrawal with CD4 count decline (Scenario F):

$\alpha_{Sj}^{T}=\alpha_{Sj}^{TF}=\alpha_{Rj}^{T1}=\alpha_{Rj}^{T2}=1$ $\{j=7,8\}$

$\varphi_{Sj}^{T}=\varphi_{Sj}^{TF}=\varphi_{Rj}^{T1}=\varphi_{Rj}^{T2}=0$ year^-1^ $\{j=7,8\}$

$\alpha_{Sj}^{T}=\alpha_{Sj}^{TF}=\alpha_{Rj}^{T1}=\alpha_{Rj}^{T2}=4$ year^-1^ $\{j=9\}$

For withdrawal with viral rebound (Scenario G):

$\alpha_{Sj}^{T}=1$ $\{j=7,8\}$

$\alpha_{Sj}^{TF}=\alpha_{Rj}^{T1}=\alpha_{Rj}^{T2}=1$ $\{j=1..8\}$

$\varphi_{Sj}^{T}=0$ year^-1^ $\{j=7,8\}$

$\varphi_{Sj}^{TF}=\varphi_{Rj}^{T1}=\varphi_{Rj}^{T2}=0$ year^-1^ $\{j=1..8\}$

$\alpha_{Sj}^{T}=\alpha_{Sj}^{TF}=\alpha_{Rj}^{T1}=\alpha_{Rj}^{T2}=4$ year^-1^ $\{j=9\}$

**Pessimistic sexual behaviour assumptions**

For the pessimistic behaviour change scenario for AIDS patients (Scenario 2), $K_{i}$ used in equations (8) and (23) above, is changed to:

$K_{i}=0$ $\left\{i=1\right\}$ $K_{i}=1-\theta_{3}-\theta_{4}$ $\left\{i=2\right\}$ $K_{i}=\theta_{3}$ $\left\{i=3\right\}$ $K_{i}=\theta_{4}$ $\left\{i=4\right\}$

Therefore AIDS patients can move to sexual activity group 2 as well as groups 3 and 4.

For the pessimistic behaviour change scenario for pre-AIDS and AIDS patients (Scenario 4), $\Lambda_{i}$ and $\tau_{i}$ used in equations (6) and (23) respectively, are changed to:

$\Lambda_{i}=\gamma_{Sj}I_{Sij}^{U}$ $\left\{i=1..4,j=10\right\}$

$\tau_{i}=\gamma_{Rj}I_{R1aij}^{U}$ $\left\{i=1..4,j=10\right\}$

Therefore there is no behaviour change upon initiating treatment for pre-AIDS patients – their sexual activity class does not change.
