# Supplementary material for: Modelling the Impact of Antiretroviral Use in Resource-Poor Settings
Source: PLoS Med. 2006 Mar 14;3(4):e124. doi: 10.1371/journal.pmed.0030124 (PMC1395349; doi:10.1371/journal.pmed.0030124)
Supplement: Supplementary file 2 [file pmed.0030124.st001.doc]

**Table S1** Descriptions and adopted values of all model parameters, with sources. The baseline values provided were used for model runs in Figure 2 and the Latin Hypercube Sampling (LHS) ranges were used for the uncertainty analyses employed in Figures 3 and 4.

| **Symbol** | **Baseline value (range)** | **Parameter Description** | **Sources** |
| --- | --- | --- | --- |
| **Behavioural parameters** | | | |
| $c_{i}$ | 152.8,13.6,0.5,0.2 partners per year  ($i$= 1,2,3,4 respectively) | Rate of partner change per year for individuals in sexual activity class $i$ | Calibrated to Malawi antenatal clinic prevalence data [1] |
| $\theta_{i}$ | 0.1%,26%,59%,15%  ($i$= 1,2,3,4 respectively) | Proportion recruited into sexual activity class $i$ |  |
| $\varepsilon$ | 0.37 | Pattern of mixing (0= fully assortative, 1= random) |  |
| **Biological parameters** | | | |
| $\beta_{S}^{U}$ | 0.03 | Transmission probability per partnership from an $I_{S}^{U}$individual during the incubation period | [2] |
| $a$ | $a=18$ $\begin{matrix} \beta_{S1}^{U}=0.54,\beta_{S2..9}^{U}=0.03 \\ \beta_{S10}^{U}=0.27,\beta_{S11}^{U}=0.54 \end{matrix}$ | Relative transmission probabilities per partnership for $I_{Sj}^{U}$ | [3,4,5,6] |
| $\frac{1}{\varphi_{S1}^{U}}$ | 1.5 months | Duration of primary infection stage for $I_{S}^{U}$ | [4,7,8,9,10] |
| $\frac{1}{\varphi_{S2..9}^{U}}$ | 1 year | Duration of incubation stage (8 years in total, from stages 2 to 9) for $I_{S}^{U}$ | [11,12,13] |
| $\frac{1}{\varphi_{S10}^{U}}$ | 1 year | Duration of pre-AIDS stage for $I_{S}^{U}$ | [14,15,16,17] |
| $\nu$ | 9 months | Duration before death with AIDS (all infection categories) | [13,16,18,19,20,21,22,23,24,25] |
| **Treatment parameters** | | | |
| $\gamma_{S1..9}$, $\gamma_{R1..9}$ | 0 | Rate of treatment uptake for $I_{S}^{U}$ and all $I_{R}^{U}$ during primary infection and incubation | Experimental values (changed in treatment scenarios) |
| $\gamma_{S10}$, $\gamma_{R10}$ | 12 year^-1^ | Rate of treatment uptake for $I_{S}^{U}$and all $I_{R}^{U}$ during pre-AIDS |  |
| $\gamma_{S11}$, $\gamma_{R11}$ | 12 year^-1^ | Rate of treatment uptake for $I_{S}^{U}$and all $I_{R}^{U}$ during AIDS |  |
| $\kappa$ | 40%  LHS range: 20-60% | Fraction of treated patients with viral rebound developing resistance at each treatment stage | [26,27] |
| $Z$ | 2%  LHS range: 1-5% | Fraction of treated patients who drop-out after each treatment stage due to side effects | [28] |
| $Z_{P}$ | 5% - best case scenario  15% - worst case scenario | Fraction of AIDS patients initiating ART who drop out due to side effects during the ‘initial’ phase | [28] |
| $f$ | 10% - best case scenario  30% - worst case scenario | Fraction of treated patients who suffer viral rebound at each treatment stage | [23,29,30,31] |
| $f_{P}$ | 30% - best case scenario  50% - worst case scenario | Fraction of AIDS patients initiating ART who develop viral rebound or failure to suppress during the ‘initial’ phase | [30,32,33,34] |
| $P$ | 2  (average duration: 6 months) | Exit rate from ‘initial’ phase for patients first starting ART | [34,35,36] |
| $\nu_{SP}^{T}$ | 10% - best case scenario  20% - worst case scenario | Fraction of AIDS patients initiating ART who die during the ‘initial’ phase | [35,37] |
| $\varphi_{S1..8}^{T}$ | 1 (average duration before progression to AIDS: 8 years) | Rate of transfer to next treatment stage, successful viral suppression | [30] |
| $\varphi_{S9}^{T}$ | 0 | Rate of transfer to next treatment stage, successful viral suppression | All patients die of AIDS at rate $\nu$ |
| $\varphi_{S1..9}^{TF}$, $\varphi_{R1..9}^{T1}$,$\varphi_{R1..9}^{T2}$ | $x\varphi_{Sj}^{T}$ $x$=1.25 | Rate of transfer to next treatment stage, treatment failure (duration before AIDS 80% of that for successful treatment) | [29,30] |
| $\varphi_{S1..9}^{UF}$, $\varphi_{R1..9}^{\vert U2}$ | $y\varphi_{Sj}^{T}$ $y$=5 | Rate of transfer to next treatment stage after removal of ART (duration before AIDS 20% of that for successful treatment ) | [38,39] |
| $\varphi_{R1..11}^{U1}$ | $\varphi_{S1..11}^{U}$ | Infection progression rate for untreated infections with primary (transmitted) resistance $I_{R1a}^{U}$(not $I_{R1b}^{U}$) | [38,39] |
| $\beta_{S1..8}^{T}$ | $w\beta_{S10}^{U}$ $w$= 2%  LHS range: 1%-10% | Transmission probability per partnership for $I_{Sj}^{T}$ | [40,41] |
| $\beta_{S9}^{T}$ | $w\beta_{S11}^{U}$ $w$= 2%  LHS range: 1%-10% | Transmission probability per partnership for $I_{Sj}^{T}$ | [40,41] |
| $\beta_{S1..8}^{TF}$,$\beta_{R1..8}^{T}$ | $z\beta_{S10}^{U}$ $z$= 75%  LHS range:50%-100% | Transmission probability per partnership for $I_{Sj}^{TF}$, $I_{Rj}^{T}$ | [33,42] |
| $\beta_{S9}^{TF}$,$\beta_{R9}^{T}$ | $z\beta_{S11}^{U}$ $z$= 75%  LHS range:50%-100% | Transmission probability per partnership for $I_{Sj}^{TF}$, $I_{Rj}^{T1}$ and $I_{Rj}^{T2}$ | [33,42] |
| $\beta_{Rj}^{U1}$ | $\beta_{Sj}^{U}$ | Transmission probability per partnership for$I_{R1aj}^{U}$ | Assumes all untreated infections have equal infectiousness. |
| $\beta_{S1..8}^{UF}$,$\beta_{R1..8}^{U2}$ | $\beta_{S10}^{U}$ | Transmission probability per partnership for $I_{Sj}^{UF}$,$I_{R1bj}^{U}$ and $I_{Rj}^{U2}$ |  |
| $\beta_{S9}^{UF}$,$\beta_{R9}^{U2}$ | $\beta_{S11}^{U}$ | Transmission probability per partnership for $I_{Sj}^{UF}$,$I_{R1bj}^{U}$ and $I_{Rj}^{U2}$ |  |
| $\omega_{U}^{2}$ | 5%  LHS range: 0-10% | Probability that an individual with secondary (acquired) resistance and not undergoing treatment transmits resistant rather than sensitive virus | [43,44] |
| $\omega_{T}^{2}$ | 30%  LHS range: 20-40% | Probability that an individual with secondary (acquired) resistance and undergoing treatment transmits resistant virus rather than sensitive virus | [42] |
| $\omega_{U1}^{1}$ | 100%  LHS range: 75-100% | Probability that an individual with primary (transmitted) resistance and primary (acute) infection, not undergoing treatment, transmits resistant rather than sensitive virus | [45,46] |
| $\omega_{U2..11}^{1}$ | 5%  LHS range: 0-10% | Probability that an individual with primary (transmitted) resistance and not undergoing treatment transmits resistant virus, all other stages of infection | [47,48] |
| $\omega_{T}^{1}$ | 100% | Probability that an individual with primary (transmitted) resistance and undergoing treatment transmits resistant rather than sensitive virus | Model assumption. |
| $\alpha_{S1..9}^{T}$,$\alpha_{S1..9}^{TF}$ $\alpha_{R1..9}^{T1}$,$\alpha_{R1..9}^{T2}$ | 0 | Baseline rate of withdrawal of failing therapy (due to primary or secondary resistance or other viral rebound) | Experimental values. |
| $\Omega$ | 5,000 | Programme capacity (i.e. maximum number that can be treated at any point in time) | Experimental values. |
| **Demographic parameters** | | | |
| $\chi$ | 0.03 | Entry rate into susceptible population | Set to produce a growing population in absence of HIV epidemic. |
| $1/\mu$ | 35 years | Duration of time individuals stay in model population | Models adults aged 15-49 years |
| $N_{0}$ | 1,000,000 | Population size at $t=0$ | Experimental value. |

$i$ represents sexual activity classes 1..4

$j$ represents stages of HIV infection 1..11

**References**

1	UNAIDS (2002) Epidemiological Fact Sheets: Malawi. Geneva, UNAIDS.

2	Baggaley RF, Boily MC, White RG, Alary M (2004) Systematic review of HIV-1 transmission probabilities in the absence of antiretroviral therapy. UNAIDS report.

3	Jacquez JA, Koopman JS, Simon CP, Longini IM, Jr. (1994) Role of the primary infection in epidemics of HIV infection in gay cohorts. J Acquir Immune Defic Syndr 7: 1169-1184.

4	Daar ES, Moudgil T, Meyer RD, Ho DD (1991) Transient high levels of viremia in patients with primary human immunodeficiency virus type 1 infection. N Engl J Med 324: 961-964.

5	Piatak M, Jr., Saag MS, Yang LC, Clark SJ, Kappes JC, et al. (1993) High levels of HIV-1 in plasma during all stages of infection determined by competitive PCR. Science 259: 1749-1754.

6	Quinn TC (1997) Acute primary HIV infection. JAMA 278: 58-62.

7	Vanhems P, Hirschel B, Phillips AN, Cooper DA, Vizzard J, et al. (2000) Incubation time of acute human immunodeficiency virus (HIV) infection and duration of acute HIV infection are independent prognostic factors of progression to AIDS. J Infect Dis 182: 334-337.

8	Niu MT, Bethel J, Holodniy M, Standiford HC, Schnittman SM (1998) Zidovudine treatment in patients with primary (acute) human immunodeficiency virus type 1 infection: a randomized, double-blind, placebo-controlled trial. DATRI 002 Study Group. Division of AIDS Treatment Research Initiative. J Infect Dis 178: 80-91.

9	Clark SJ, Saag MS, Decker WD, Campbell-Hill S, Roberson JL, et al. (1991) High titers of cytopathic virus in plasma of patients with symptomatic primary HIV-1 infection. N Engl J Med 324: 954-960.

10	Horsburgh CR, Jr., Ou CY, Jason J, Holmberg SD, Longini IM, Jr., et al. (1989) Duration of human immunodeficiency virus infection before detection of antibody. The Lancet 2: 637-640.

11	Crampin AC, Floyd S, Glynn JR, Sibande F, Mulawa D, et al. (2002) Long-term follow-up of HIV-positive and HIV-negative individuals in rural Malawi. AIDS 16: 1545-1550.

12	Morgan D, Whitworth J (2001) The natural history of HIV-1 infection in Africa. Nat Med 7: 143-145.

13	Sewankambo NK, Gray RH, Ahmad S, Serwadda D, Wabwire-Mangen F, et al. (2000) Mortality associated with HIV infection in rural Rakai District, Uganda. AIDS 14: 2391-2400.

14	Fauci AS, Pantaleo G, Stanley S, Weissman D (1996) Immunopathogenic mechanisms of HIV infection. Ann Intern Med 124: 654-663.

15	Connor RI, Mohri H, Cao Y, Ho DD (1993) Increased viral burden and cytopathicity correlate temporally with CD4+ T-lymphocyte decline and clinical progression in human immunodeficiency virus type 1-infected individuals. J Virol 67: 1772-1777.

16	Boily MC, Lowndes C, Alary M (2002) The impact of HIV epidemic phases on the effectiveness of core group interventions: insights from mathematical models. Sex Transm Infect 78 Suppl 1: i78-90.

17	Aalen OO, Farewell VT, De Angelis D, Day NE, Gill ON (1999) New therapy explains the fall in AIDS incidence with a substantial rise in number of persons on treatment expected. AIDS 13: 103-108.

18	Kitayaporn D, Tansuphaswadikul S, Lohsomboon P, Pannachet K, Kaewkungwal J, et al. (1996) Survival of AIDS patients in the emerging epidemic in Bangkok, Thailand. J Acquir Immune Defic Syndr Hum Retrovirol 11: 77-82.

19	Mulder DW, Nunn AJ, Kamali A, Nakiyingi J, Wagner HU, et al. (1994) Two-year HIV-1-associated mortality in a Ugandan rural population. The Lancet 343: 1021-1023.

20	Morgan D, Malamba SS, Orem J, Mayanja B, Okongo M, et al. (2000) Survival by AIDS defining condition in rural Uganda. Sex Transm Infect 76: 193-197.

21	Gail MH, Tan WY, Pee D, Goedert JJ (1997) Survival after AIDS diagnosis in a cohort of hemophilia patients. Multicenter Hemophilia Cohort Study. J Acquir Immune Defic Syndr Hum Retrovirol 15: 363-369.

22	Chequer P, Hearst N, Hudes ES, Castilho E, Rutherford G, et al. (1992) Determinants of survival in adult Brazilian AIDS patients, 1982-1989. The Brazilian State AIDS Program Co-Ordinators. AIDS 6: 483-487.

23	Nagelkerke NJ, Jha P, de Vlas SJ, Korenromp EL, Moses S, et al. (2002) Modelling HIV/AIDS epidemics in Botswana and India: impact of interventions to prevent transmission. Bull World Health Organ 80: 89-96.

24	Robinson NJ, Mulder D, Auvert B, Whitworth J, Hayes R (1999) Type of partnership and heterosexual spread of HIV infection in rural Uganda: results from simulation modelling. Int J STD AIDS 10: 718-725.

25	Wood E, Braitstein P, Montaner JS, Schechter MT, Tyndall MW, et al. (2000) Extent to which low-level use of antiretroviral treatment could curb the AIDS epidemic in sub-Saharan Africa. The Lancet 355: 2095-2100.

26	Gallego O, Ruiz L, Vallejo A, Clotet B, Leal M, et al. (2002) Rate of Virological Treatment Failure and Frequencies of Drug Resistance Genotypes among Human Immunodeficiency Virus-Positive Subjects on Antiretroviral Therapy in Spain. J Clin Microbiol 40: 3865-3866.

27	Weidle PJ, Malamba S, Mwebaze R, Sozi C, Rukundo G, et al. (2002) Assessment of a pilot antiretroviral drug therapy programme in Uganda: patients' response, survival, and drug resistance. The Lancet 360: 34-40.

28	Kumarasamy N, Solomon S, Chaguturu SK, Mahajan AP, Flanigan TP, et al. (2003) The safety, tolerability and effectiveness of generic antiretroviral drug regimens for HIV-infected patients in south India. Aids 17: 2267-2269.

29	Ledergerber B, Egger M, Opravil M, Telenti A, Hirschel B, et al. (1999) Clinical progression and virological failure on highly active antiretroviral therapy in HIV-1 patients: a prospective cohort study. Swiss HIV Cohort Study. The Lancet 353: 863-868.

30	Lucas GM, Chaisson RE, Moore RD (2003) Survival in an urban HIV-1 clinic in the era of highly active antiretroviral therapy: a 5-year cohort study. J Acquir Immune Defic Syndr 33: 321-328.

31	Blower SM, Gershengorn HB, Grant RM (2000) A tale of two futures: HIV and antiretroviral therapy in San Francisco. Science 287: 650-654.

32	Rastegar DA, Fingerhood MI, Jasinski DR (2003) Highly active antiretroviral therapy outcomes in a primary care clinic. AIDS Care 15: 231-237.

33	Deeks SG, Hecht FM, Swanson M, Elbeik T, Loftus R, et al. (1999) HIV RNA and CD4 cell count response to protease inhibitor therapy in an urban AIDS clinic: response to both initial and salvage therapy. AIDS 13: F35-43.

34	Moore RD, Chaisson RE (1999) Natural history of HIV infection in the era of combination antiretroviral therapy. AIDS 13: 1933-1942.

35	Tassie JM, Szumilin E, Calmy A, Goemaere E (2003) Highly active antiretroviral therapy in resource-poor settings: the experience of Medecins Sans Frontieres. AIDS 17: 1995-1997.

36	Johnson L, Dorrington R (2002) The demographic and epidemiological impact of HIV/AIDS treatment and prevention programmes: an evaluation based on the ASSA2000 model. Paper presented at the Demographic Association of Southern Africa Conference, Cape Town, 26-27 September 2002.

37	Lee LM, Karon JM, Selik R, Neal JJ, Fleming PL (2001) Survival after AIDS diagnosis in adolescents and adults during the treatment era, United States, 1984-1997. JAMA 285: 1308-1315.

38	Miller V, Sabin C, Hertogs K, Bloor S, Martinez-Picado J, et al. (2000) Virological and immunological effects of treatment interruptions in HIV-1 infected patients with treatment failure. AIDS 14: 2857-2867.

39	Deeks SG, Wrin T, Liegler T, Hoh R, Hayden M, et al. (2001) Virologic and immunologic consequences of discontinuing combination antiretroviral-drug therapy in HIV-infected patients with detectable viremia. N Engl J Med 344: 472-480.

40	Musicco M, Lazzarin A, Nicolosi A, Gasparini M, Costigliola P, et al. (1994) Antiretroviral treatment of men infected with human immunodeficiency virus type 1 reduces the incidence of heterosexual transmission. Italian Study Group on HIV Heterosexual Transmission. Arch Intern Med 154: 1971-1976.

41	Perelson AS, Essunger P, Cao Y, Vesanen M, Hurley A, et al. (1997) Decay characteristics of HIV-1-infected compartments during combination therapy. Nature 387: 188-191.

42	Leigh Brown AJ, Frost SD, Mathews WC, Dawson K, Hellmann NS, et al. (2003) Transmission fitness of drug-resistant human immunodeficiency virus and the prevalence of resistance in the antiretroviral-treated population. J Infect Dis 187: 683-686.

43	Little SJ, Daar ES, D'Aquila RT, Keiser PH, Connick E, et al. (1999) Reduced antiretroviral drug susceptibility among patients with primary HIV infection. JAMA 282: 1142-1149.

44	Wainberg M, Friedland G (1998) Public health implications of antiretroviral therapy and HIV drug resistance. JAMA 279: 1977-1983.

45	Hance AJ, Lemiale V, Izopet J, Lecossier D, Joly V, et al. (2003) Transmission fitness of drug-resistant human immunodeficiency virus type 1 populations after treatment interruption in patients failing antiretroviral-treated population. J Virol 187: 6410-6417.

46	Yerly S, Jost S, Telenti A, Flepp M, Kaiser L, et al. (2002) Transmission of drug resistance: impact of primary and chronic HIV infection. Swiss HIV Cohort Study. Antivir Ther 7: S150.

47	Hance AJ, Lemiale V, Izopet J, Lecossier D, Joly V, et al. (2001) Changes in human immunodeficiency virus type 1 populations after treatment interruption in patients failing antiretroviral therapy. J Virol 75: 6410-6417.

48	Nicastri E, Sarmati L, d'Ettorre G, Palmisano L, Parisi SG, et al. (2003) Replication capacity, biological phenotype, and drug resistance of HIV strains isolated from patients failing antiretroviral therapy. J Med Virol 69: 1-6.
